# Risk factors for sustained virological non-suppression among children and adolescents living with HIV in Zimbabwe and Malawi: a secondary data analysis

**DOI:** 10.1186/s12887-022-03400-4

**Published:** 2022-06-11

**Authors:** Christi Jackson, Andrea M. Rehman, Grace McHugh, Carmen Gonzalez-Martinez, Lucky G. Ngwira, Tsitsi Bandason, Hilda Mujuru, Jon O. Odland, Elizabeth L. Corbett, Rashida A. Ferrand, Victoria Simms

**Affiliations:** 1 Department of Clinical Research, London School of Hygiene & Tropical Medicine, London, UK; 2 MRC International Statistics and Epidemiology Group, Department of Infectious Disease Epidemiology, London School of Hygiene & Tropical Medicine, London, UK; 3 Biomedical Research and Training Institute, Harare, Zimbabwe; 4 Malawi-Liverpool-Wellcome Trust Clinical Research Programme, Blantyre, Malawi; 5 Department of Clinical Sciences, Liverpool School of Tropical Medicine, Liverpool, UK; 6 Department of Paediatrics, University of Zimbabwe, Harare, Zimbabwe; 7 School of Health Systems and Public Health, Faculty of Health Sciences, University of Pretoria, Pretoria, South Africa; 8 Department of General Hygiene I.M. Sechenov First, Moscow State Medical University (Sechenov University), Moscow, Russia

**Keywords:** Adolescent, Antiretroviral therapy, Chronic lung disease, HIV viral load, Resistance, Viral non-suppression

## Abstract

**Background:**

We investigated risk factors for sustained virological non-suppression (viral load ≥ 1000 copies/ml on two tests 48 weeks apart) among children and adolescents accessing HIV care in public sector clinics in Harare, Zimbabwe and Blantyre, Malawi.

**Methods:**

Participants were enrolled between 2016 and 2019, were aged 6–19 years, living with HIV, had chronic lung disease (FEV z-score < -1) and had taken antiretroviral therapy (ART) for at least six months. We used multivariate logistic regression to identify risk factors for virological non-suppression after 48 weeks, among participants who were non-suppressed at enrolment.

**Results:**

At enrolment 258 participants (64.6%) were on first-line ART and 152/347 (43.8%) had virological non-suppression. After 48 weeks 114/313 (36.4%) were non-suppressed. Participants non-suppressed at baseline had almost ten times higher odds of non-suppression at follow-up (OR = 9.9, 95%CI 5.3–18.4, *p* < 0.001). Of those who were non-suppressed at enrolment, 87/136 (64.0%) were still non-suppressed at 48 weeks. Among this group non-suppression at 48 weeks was associated with not switching ART regimen (adjusted OR = 5.55; 95%CI 1.41–21.83); *p* = 0.014) and with older age. Twelve participants switched regimen in Zimbabwe and none in Malawi.

**Conclusions:**

Viral non-suppression was high among this group and many with high viral load were not switched to a new regimen, resulting in continued non-suppression after 48 weeks. Further research could determine whether improved adherence counselling and training clinicians on regimen switches can improve viral suppression rates in this population.

**Trial registration:**

Secondary cohort analysis of data from BREATHE trial (Clinicaltrials.gov NCT02426112).

## Background

The scale-up of paediatric antiretroviral therapy (ART) programmes in sub-Saharan Africa has resulted in dramatically improved survival of children with HIV and a growing population of adolescents living with HIV. Several studies have shown that virological outcomes of children and adolescents are worse than those of adults. The most recent Population Based HIV Impact Assessment (PHIA) survey in Zimbabwe, which was done from 2015–16 [1] estimated that 68.8% of 0–14 year-olds and 82.4% of 15–24 year-olds on ART were virally suppressed, compared to 90.3% of adults on ART in the same period. In the Malawi PHIA survey, also done in 2015–16 viral 57.9% of children aged 0–14 years and 81.2% of 15–24 year olds on ART were virally suppressed, while 91.3% adults on ART had viral suppression.

The national ART guidelines for Zimbabwe [2] and Malawi [3] are closely aligned to WHO recommendations [4]. Since 2016, both countries have adopted a “treat all” approach, meaning that anyone with HIV is eligible for ART, regardless of CD4 count or clinical status. Adherence counselling at treatment initiation emphasizes the need for at least 95% adherence to treatment, and both countries have defined approaches to managing children and adolescents with viral non-suppression including intensive adherence counselling and subsequent switching to second-line ART if viral suppression is not achieved by 3 months after counselling. HIV resistance testing is difficult to access in Zimbabwe and Malawi and is usually reserved for patients failing second line ART regimens.

Several studies have described failure to switch adults and children living with HIV with unsuppressed viral load or ART resistance onto second and third line regimens in sub-Saharan Africa [5, 6].

BREATHE was a randomised placebo-controlled trial to investigate whether weekly azithromycin over 48 weeks improved lung function in children and adolescents living with HIV (CALHIV) who had chronic lung disease, in Zimbabwe and Malawi[7]. Participants underwent HIV viral load testing at enrolment and at 48 weeks; a higher than expected number of trial participants remained unsuppressed 48 weeks later. We investigated risk factors associated with unsuppressed viral load at enrolment and with remaining unsuppressed at 48 weeks.

## Methods

The BREATHE trial (Clinicaltrials.gov registration NCT02426112), a double-blinded placebo controlled individually randomised trial of azithromycin for management of chronic lung disease, enrolled 347 children and adolescents aged 6–19 years with perinatally acquired HIV, who had been on ART for at least six months prior to enrolment and had a forced expiratory volume in one second (FEV1) z-score below -1 [8]. In Zimbabwe, participants were enrolled from the outpatient HIV clinic at Harare Hospital and surrounding primary care clinics in Harare. In Malawi, enrolments were done from Queen Elizabeth Hospital as well as ART clinics within Blantyre district between 2016 and 2019. Participants received weight-based weekly doses of azithromycin or placebo for 48 weeks. The primary outcome was FEV1 z-score. The results of the trial have been reported separately [9].

Participants continued to receive HIV care at their usual public-sector facilities during the trial period. When the study team identified a participant with an unsuppressed viral load, they conducted enhanced adherence counselling with the participant and guardian to identify potential barriers to adherence. A referral letter with copy of the viral load result was sent to the participant’s HIV treatment facility for potential ART regimen switch. Patient-held ART records were reviewed during study visits and any regimen changes noted, but this was not cross-checked with medical records at the treatment facility. In Zimbabwe, the study team aimed to repeat a targeted viral load 3 months after an unsuppressed viral load, while in Malawi a recommendation was made to the usual health facility to do so. Access to viral load monitoring was not always available.

All analyses were carried out in Stata v15 or v16 (StataCorp, College Station, TX, USA). The point prevalence of unsuppressed HIV viral load (defined as ≥ 1000 copies/mL) was calculated at enrolment and at 48-weeks. The proportion with viral non-suppression at baseline and at 48 weeks.by socio-demographic variables, duration and type of ART regimen (first, second or third line ART), dosing schedule (once or twice daily) and baseline CD4 count was calculated.

Crude odds ratios with 95% confidence intervals and *p*-values were also calculated for the association between these variables and viral non-suppression at baseline and at 48 weeks. Viral load was compared at baseline and after 48 weeks.

Logistic regression was used to investigate risk factors that were associated with viral non-suppression at 48 weeks in the subgroup of participants who had a viral load ≥ 1000 copies/mL at enrolment. As well as socio-demographic and ART-related factors, an unscheduled study visit and adherence to the study drug were included in this analysis. Adherence to the study drug was defined as not missing more than a mean of two doses per 12-week period during the 48 weeks of the intervention [10]. As a post hoc sensitivity analysis, the investigation of factors associated with viral non-suppression at 48 weeks was repeated in the Zimbabwe group only.

Exposure variables associated with the outcome with *p*-values < 0.1 in univariate analysis were included in the multivariate analysis and age group, sex and country were adjusted for a priori.

## Results

A total of 347 children and adolescents were enrolled in the BREATHE trial, 241 (69.5%) in Zimbabwe and 106 in Malawi (Table 1). The median age at enrolment was 15 (IQR 12.7–17.7) years. At enrolment 285 participants (83.1%) were attending school. Of those not attending school, 94.8% were aged 16–19 years. More than half of participants (*n* = 197, 58.1%) had repeated a school grade at least once. Most of the participants’ mothers (*n* = 213/302, 70.5%) had completed secondary school as their highest level of education, 22 (7.3%) had never been to school, and 8 (2.7%) had attended tertiary education. Education levels were higher in Zimbabwe, with 88.1% of mothers having secondary or higher education, compared to 43.6% in Malawi.

**Table 1:** Prevalence of viral load (VL) ≥ 1000 copies/mL at enrolment and 48 weeks by participant characteristics

Characteristic		All participants		VL ≥ 1000 copies/mL at enrolment		VL ≥ 1000 copies/mL at 48 weeks	
		N	%	N	%	N	%
	Total	347		151/345	43.8%	114/315	36.2%
Country	Zimbabwe	241	69.5%	100/241	41.5%	79/224	35.3%
	Malawi	106	30.5%	51/104	49.0%	35/91	38.5%
Trial arm	Control	174	50.1%	80/174	46.0%	56/154	36.4%
	Intervention	173	49.9%	71/171	41.5%	58/161	36.0%
Sex	Male	177	51.0%	82/177	46.3%	53/166	31.9%
	Female	170	49.0%	69/168	41.1%	61/149	40.9%
Age at enrolment, years	6–10	47	13.5%	17/47	36.2%	9/44	20.5%
	11–15	158	45.5%	69/156	44.2%	48/143	33.6%
	16–19	142	40.9%	65/142	45.8%	57/128	44.5%
ART regimen at enrolment	1st line (NNRTI)	258	74.6%	110/256	43.0%	83/236	35.2%
	2nd line (PI)	86	24.9%	41/86	47.7%	31/76	40.8%
	3rd line	2	0.6%	0/2	0.0%	0/2	0.0%
	Unknown	1					
Timing of regimen dosing	Once daily	156	45.0%	71/156	45.5%	61/141	43.3%
	Twice daily	191	55.0%	80/189	42.3%	53/174	30.5%
Duration on ART	< 2y	33	9.8%	7/32	21.9%	7/30	23.3%
	2-5y	130	38.6%	56/129	43.4%	40/120	33.3%
	6-10y	126	37.39%	58/126	46.0%	48/115	41.7%
	> 11y	48	14.24%	24/48	50.0%	18/41	43.9%
	Missing	10					
Baseline viral load, copies/mL	< 50	105	30.4%	-		11/94	11.7%
	50–999	89	25.8%	-		16/83	19.3%
	≥ 1000	151	43.8%	-		87/136	64.0%
	Missing	2					
Baseline CD4 count, cells/µL	< 200	34	9.8%	29/34	85.3%	18/31	58.1%
	200–499	96	27.7%	54/95	56.8%	49/87	56.3%
	≥ 500	217	62.5%	68/216	31.5%	47/197	23.9%
Previous history of TB	No	249	72.0%	103/247	41.7%	75/223	33.6%
	Yes	97	28.0%	48/97	49.5%	39/91	42.9%
	Missing	1					
Currently in school	Yes	285	83.1%	120/284	42.3%	91/262	34.7%
	No	58	16.9%	30/58	51.7%	23/50	46.0%
	Missing	4					
Ever repeated a grade in school	No	142	41.9%	57/142	40.1%	40/133	30.1%
	Yes	197	58.1%	91/196	46.4%	73/176	41.5%
	Missing	8					
Education level of mother	No schooling	22	7.3%	12/22	54.5%	44,186	57.1%
	Primary	59	19.5%	28/58	48.3%	22/50	44.0%
	Secondary	213	70.5%	80/212	37.7%	63/200	31.5%
	Tertiary	8	2.7%	4/8	50.0%	2/8	25.0%
	Missing	45					
Adherence	Adherent	249	73.5%	102/249	41.0%	83/244	34.0%
	Non-adherent	90	26.6%	44/90	50.0%	27/64	42.2%
	Missing	8		5/8	62.5%	4/7	57.1%
Unscheduled clinical visit for illness during follow-up	No	274	84.5%	104/272	38.2%	86/266	32.3%
	Yes	50	15.5%	34/50	68.0%	28/49	57.1%
	Missing	23					

The median age at ART initiation was 8.4 (IQR 5.7–11.6) years, and median duration on ART was 6.2 (IQR 3.9–8.4) years. Most participants (*n* = 258, 74.6%) were on a non-nucleoside reverse transcriptase inhibitor (NNRTI)-based regimen at enrolment, 86 (24.9%) were on a protease inhibitor (PI)-based regimen and two were on a third line regimen containing an integrase inhibitor. Baseline viral load results were missing for two participants. Viral load at enrolment was < 1000 copies/mL for 194 (56.2%) participants, of which 105(54.1%) were < 50 copies and 89 (45.9%) were 50–999 copies/mL. Amongst the 151 participants with a viral load ≥ 1000 copies/mL at enrolment, the median time since ART initiation was 6.6 years (IQR 4.2–8.7 years).

Viral load results at enrolment and 48 weeks were available for 313/347 (90.2%) participants. The proportion of participants with viral loads ≥ 1000 copies/mL was 43.5% (*n* = 136) at enrolment and 36.4% (*n* = 114) at follow-up. Less than half of participants (*n* = 150, 47.9%) were virally suppressed at both timepoints (Table 2). Another 27 participants were suppressed at enrolment but had a raised viral load ≥ 1000 copies/mL at 48 weeks (20/27 were female). Of the 136 participants with viral non-suppression at enrolment, 49 had viral suppression at follow-up. Notably, 87 participants (27.8% of the total group, 64.0% of those with a raised baseline viral load) had unsuppressed viral loads both at enrolment and follow-up.

**Table 2:** Viral suppression at enrolment compared to 48 weeks, among those with both valid results

		**VL at 48 weeks (copies/mL)**		
		** < 1000**	** ≥ 1000**	**Total**
**VL at enrolment (copies/mL)**	** < 1000**	150 (47.9%)	27 (8.6%)	177 (56.5%)
	** ≥ 1000**	49 (15.7%)	87 (27.8%)	136 (43.5%)
	**Total**	199 (63.6%)	114 (36.4%)	313 (100%)

Participants with a viral load ≥ 1000 copies/mL at baseline had almost ten times higher odds of unsuppressed viral load at follow-up, compared to those with a viral load < 1000 copies at enrolment (OR = 9.9, 95% CI 5.3–18.4, *p* < 0.001). There was weak evidence that low-level viraemia at enrolment (50–999 copies/mL) was associated with increased odds of viral load > 1000 copies/mL at follow-up (OR = 1.8, 95% CI 0.78–4.17, *p* = 0.163).

During the study period 29 participants switched ART regimen, all of them from Zimbabwe (Table 3). Of these, 14 changed from a first line NNRTI regimen to a second line PI regimen, 10 changed to an alternative first line, 4 to an alternative second line, and 1 reported a change from a PI to an NNRTI regimen. Reason for regimen switch was not captured. Out of 151 participants with viral load > 1000 at enrolment, 110 were on an NNRTI regimen at enrolment and 13/110 switched to PI, while 41 were on a PI regimen at enrolment and 1/41 switched to NNRTI. In Zimbabwe, 13/67 participants (19.4%) with viral load ≥ 1000 copies/mL at enrolment switched from NNRTI regimens to PI. None of the 41 participants on PI-regimens with unsuppressed baseline viral load were changed to third line regimens.

**Table 3:** Changes in ART regimen anchor drug between enrolment and 48 weeks

Anchor drug at enrolment		**Anchor drug at 48 weeks**					
		**NNRTIs**		**PIs**		**INSTI**	**Total**
		**NVP**	**EFV**	**LPV**	**ATV**		
**NNRTIs**	**NVP**	122	6	3	1	0	**132**
	**EFV**	1	115	3	7	0	**126**
**PIs**	**LPV**	0	1	28	1	0	**30**
	**ATV**	0	0	0	56	0	**56**
**INSTI**		0	0	0	0	2	**2**
**Total**		**123**	**122**	**34**	**65**	**2**	**346**

In univariate analysis, there was weak evidence that virological non-suppression at enrolment was associated with longer duration on ART, compared to those who had been on ART for less than 2 years (Table 4). This association remained the same after adjusting for age group, sex and country. After adjusting for age, sex and duration on ART there was weak evidence that participants in Malawi were more likely to be non-suppressed (aOR = 1.56 (95% CI 0.94–2.60; *p* = 0.09). No other factors were associated with baseline virological non-suppression.

**Table 4:** Risk factors for viral non-suppression at enrolment

Variable	Categories	VL > 1000 at enrolment		Univariable (*N* = 345)		Multivariable (*N* = 335)	
		**N**	**%**	**Crude OR (95% CI)**	**p**	**Adjusted OR (95% CI)**	**p**
**Total**		151	43.8				
Country	Zimbabwe	100	41.5	1	0.20	1	0.09
	Malawi	51	49.0	1.36 (0.85, 2.16)		1.56 (0.94, 2.60)	
Sex	Male	82	46.3		0.33	1	
	Female	69	41.1	0.81 (0.53 1.24)		0.75 (0.48, 1.16)	0.20
Age group	6-10y	17	36.2	1	0.51	1	0.63
	11-15y	69	44.2	1.40 (0.71, 2.75)		1.28 (0.63, 2.58)	
	16-19y	65	45.8	1.49 (0.75, 2.95)		1.42 (0.69, 2.92)	
ART regimen at enrolment	NNRTI	110	43.0	1	0.56		
	PI/3^rd^ line	41	46.6	1.16 (0.71, 1.89)			
Regimen timing at enrolment	Once daily	71	45.5	1	0.55		
	Twice daily	80	42.3	0.88 (0.57, 1.35)			
Duration on ART, years (*N* = 335)	< 2	7	21.9	1	0.07	1	0.08
	2–5	56	43.4	2.74 (1.09, 6.91)		2.96 (1.17, 7.46)	
	6–9	58	46.0	3.05 (1.20, 7.72)		3.31 (1.30, 8.42)	
	> 10	24	50.0	3.57 (1.24, 10.32)		3.51 (1.24, 9.98)	
History of TB	Yes	48	49.5	1.37 (0.85, 2.20)	0.19		
	No	103	41.7	1			
Currently in school	Yes	120	42.3	1	0.19		
	No	30	51.7	1.46 (0.83, 2.59)			
Ever repeated a grade (*N* = 338)	Yes	91	40.1	1	0.25		
	No	57	46.4	1.29 (0.83, 2.00)			
Education of mother (*N* = 300)	No schooling	12	54.6	1	0.25		
	Primary	28	48.3	0.78 (0.29, 2.10)			
	Secondary	80	37.7	0.51 (0.21, 1.23)			
	Tertiary	4	50.0	0.83 (0.16, 4.34)			

Out of 136 participants who had an unsuppressed viral load at enrolment, 87 (64.0%) still had a viral load ≥ 1000 copies/mL at 48 weeks (Table 5). In univariate analysis, participants who remained on the same ART regimen had 4 times higher odds of virological failure at follow-up compared to those that changed from a NNRTI-based to a PI-based regimen (OR 4.05, 95% CI 1.12–14.67, *p* = 0.021). Older adolescents of 16–19 years had higher odds of virological non-suppression. Unsuppressed viral load was weakly associated with the participant’s mother having less formal education and with longer duration on ART.

**Table 5:** Characteristics associated with unsuppressed viral load at 48 weeks, among participants unsuppressed enrolment

Variable	Categories	VL > 1000 at 48 weeks		Univariable (*N* = 136)		Multivariable (*N* = 109)		Multivariable, Zimbabwe (*N* = 92)	
		**N**	**%**	**Crude OR (95% CI)**	**p**	**Adjusted OR (95% CI)**	**p**	**Adjusted OR (95% CI)**	**p**
**Total**		87/136	64.0						
Regimen change	Yes	4/12	33.3	1	0.021	1	0.020	1	
	No	83/124	66.9	4.05 (1.12–14.67)		7.68 (1.38–42.63)		5.55 (1.41–21.83)	0.014
Country	Zimbabwe	59/93	63.4	1	0.85	1	0.53		
	Malawi	28/43	65.1	1.08 (0.50–2.30)		1.46 (0.45–4.71)			
Trial arm	Control	47/72	65.3	1	0.74				
	Intervention	40/64	62.5	0.89 (0.44–1.79)					
Sex	Male	46/77	59.7	1	0.24				
	Female	41/59	69.5	1.54 (0.74–3.17)					
Age at enrolment, years	6–10	9/16	56.3	1	0.031	1	0.008	1	0.07
	11–15	34/62	54.8	0.94 (0.31–2.88)		0.82 (0.22–3.12)		0.74 (0.15–3.61)	
	16–19	44/58	75.9	2.44 (0.75–7.98)		6.31 (1.25–31.93)		2.30 (0.46–11.54)	
ART regimen at enrolment	NNRTI	65/100	65.0	1	0.68				
	PI	22/36	61.1	0.85 (0.38–1.86)					
Duration on ART, years (*N* = 131)	< 2	3/7	42.9	1	0.073	1	0.24		
	2–5	29/51	56.9	1.76 (0.35–8.85)		4.23 (0.47–37.65)			
	6–9	40/52	76.9	4.44 (0.81–24.25)		8.57 (0.94–78.28)			
	> 10	14/21	66.7	2.67 (0.43–16.52)		5.75 (0.55–59.75)			
History of TB	Yes	27/44	61.4	0.85 (0.40–1.79)	0.66				
	No	60/92	65.2	1					
Currently in school	Yes	68/109	62.4	1	0.44				
	No	19/27	70.4	1.43 (0.57–3.59)					
Education of mother (*N* = 114)	No schooling	10/12	83.3	1	0.054	1	0.093		
	Primary	17/23	73.9	0.57 (0.09–3.49)		0.67 (0.06–7.73)			
	Secondary	45/75	60.0	0.30 (0.06–1.51)		0.13 (0.01–1.30)			
	Tertiary	2/4	50.0	0.29 (0.01–3.02)		0.12 (0.00–3.31)			
Study drug adherence (*N* = 131)	Adherent	61/101	60.4	1	0.20				
	Non-adherent	22/30	73.3	1.80 (0.72–4.49)					
Any unscheduled clinic visit for illness	Yes	64/102	62.8	1.24 (0.54, 2.84)	0.61				
	No	23/34	67.7	1					

After adjusting for study site, age, duration on ART and mother’s education, participants who did not switch ART regimen had more than seven times the odds of unsuppressed viral load at follow-up (adjusted OR 7.68, 95% CI 1.38–42.63; *n* = 109). After adjustment, there was strong evidence that staying on the same ART regimen was associated with unsuppressed viral load at 48 weeks (*p* = 0.020). Older age was still strongly associated with the outcome. Duration on ART was not associated with unsuppressed viral load at 48 weeks. The apparent association with mother’s education was due to confounding by country, as education was lower in Malawi and unsuppressed viral load was more prevalent there. When the analysis was repeated with Zimbabwe participants only, there was still strong evidence that not switching regimen was associated with unsuppressed viral load at 48 weeks and weak evidence of association with age.

## Discussion

We found that viral non-suppression was high among CALHIV with chronic lung disease, and that many with high viral load were not switched to a new regimen, resulting in continued non-suppression after 48 weeks.

At enrolment into the BREATHE trial, only 56.2% of participants, who had all been on ART for at least six months, had a suppressed viral load. This stands in contrast to reported levels of viral suppression in adults in the same countries: UNAIDS data shows 87% of adults on ART in Zimbabwe and 89% in Malawi were virally suppressed in 2019 [11], and also lower than reported levels for CALHIV in the wider population (68–82% in Zimbabwe [1] and 57–81% in Malawi [12]). The study participants had all been diagnosed with chronic lung disease, and almost a third had a history of tuberculosis. Chronic lung disease is more likely to occur in children who started ART late, and is likely to persist even after ART initiation and subsequent viral suppression [13]. However, it is possible that ongoing viral replication may increase the risk of recurrent viral and bacterial infections of the respiratory tract as well as chronic pulmonary inflammation. These may in turn have long-term sequalae such as bronchiectasis and obliterative bronchiolitis [14], which could explain the higher prevalence of HIV viral non-suppression at study enrolment. Additionally, medications used by participants for their chronic lung disease contribute to an increased pill burden, which is associated with poorer adherence, and could also explain the higher levels of viral non-suppression.

Older adolescents were more likely to have unsuppressed viral load. This aligns with findings from other studies [15], which showed that adherence levels are higher in younger children compared to older children and adolescents. A possible reason is that younger children are more likely to be supervised by a caregiver and therefore have higher adherence, whereas adolescents are often perceived to be old enough to take their treatment unsupervised. They may however not achieve optimal adherence levels due to forgetfulness, pill fatigue, or because they lack the cognitive maturity to appreciate the long-term benefits of adherence [16].

Less than half of participants (47.9%) were virally suppressed at both time points. Participants who had a low-level viraemia (VL 50–999 copies/mL) at enrolment were more likely to progress to a viral load ≥ 1000 copies/mL by 48 weeks, compared to those who were fully suppressed at baseline, although this finding was not statistically significant (OR 1.8, *p* = 0.16). This is in line with findings from previous studies, including a study by the Antiretroviral Therapy Cohort Collaboration (ART-CC) [17], which showed that low-level viraemia (defined as 200–499 copies/mL) was associated with subsequent virological failure (adjusted hazard ratio 3.97, 95% CI 3.05–5.17). The WHO guidelines that were in place during the study period^[4]^ used the 1000 copies/ml cut-off to define viral non-suppression, which was pragmatic with no guidance on how to address low-level viraemia. This has since been updated, and the WHO consolidated guidelines of July 2021 [18] advise enhanced adherence counselling and additional viral load testing to promote viral suppression.

It is not surprising that there was strong evidence for an association between staying on the same ART regimen and having unsuppressed viral load at follow-up, given that the majority of participants had been on ART for several years and could have developed resistance mutations to their current treatment. There was also strong evidence for an association of older age (16–19 years) with unsuppressed viral load at follow-up, despite this age group being more likely to have had an ART regimen change compared to younger participants. This could point to ongoing adherence problems in older adolescents, and the need for tailored adherence interventions for this group. Longer duration on ART was also associated with the outcome, possibly due to treatment fatigue and increased risk of having developed treatment resistance due to previous episodes of poor adherence.

Nearly 70% of participants who were unsuppressed at baseline and who remained on first line ART still had unsuppressed viral loads after 48 weeks. Because genotypic resistance testing was not available, it is not possible to know what proportion of these participants had resistance to first line drugs versus the proportion who had suboptimal adherence to an effective regimen, and could potentially suppress their viral load with the correct adherence support. There is accumulating evidence of ART resistance levels among adolescents in Zimbabwe. Out of 102 children and adolescents in Harare with virological failure in 2012, 67.6% had ≥ 1 clinically significant mutation [19]. In 2016, out of 726 young people aged 16–24 who received enhanced adherence counselling, 74 (10.2%) had virological failure and 72/74 had at least 1 drug resistance mutation [20]. Both Zimbabwe and Malawi country guidelines provide clear steps to follow after finding a raised viral load of ≥ 1000 copies/mL in a patient, such as enhanced adherence counselling and targeted repeat viral load testing after 3 months, with a regimen change indicated if the viral load remains unsuppressed despite improved adherence.

No regimen switches were reported in Malawi, while in Zimbabwe only 14 participants switched from a first line NNRTI to a second line PI-regimen. In both these countries, outpatient medical records include a paper health passport that is patient-held, and which the participants could bring along to study visits. It is possible that more regimen switches could have taken place and not been recorded in these health passports, or that a patient may have several health passports, but local clinical staff confirmed that it is likely that no Malawian and only few Zimbabwean participants switched to second line ART. Previous studies have described similar findings [6, 21]. It is not clear why patients are not changed to second line when they qualify, but several reasons have been postulated [6, 22, 23]. These include: a lack of expertise or confidence by the HIV care provider to diagnose virological failure and choose appropriate second line ART; reluctance to change to a regimen with a higher pill burden and potentially more side effects, since adherence is already perceived to be a challenge; poor palatability of LPV/r syrup; uncertainty of how to assess adherence; a sense of hope that the viral load will be suppressed if adherence is emphasized and the test repeated; wanting to avoid drug interactions in patients taking rifampicin for TB treatment; unavailability of viral load testing or access to results in some areas; shortages or supply problems with second line drugs; and the higher cost of second line ART [6, 22]. Solutions could include training and mentoring of staff on diagnosing and managing regimen changes; implementing new developments such as point of care viral load testing, when available and approved, so that the short turnaround time can assist with more rapid management of abnormal results; procuring new drug formulations with improved tolerability and pill burden, when available; and thorough counselling of patients and their families on the importance of adhering to the second line regimen. In rural South Africa, quality improvement and clinical mentorship led to more children being switched to second line therapies [24].

We acknowledge several study limitations. The main aim of the BREATHE trial was to assess a change in lung function in response to azithromycin treatment, rather than virological outcomes [9]. Some information that would have been useful for this secondary analysis was not collected, including ART adherence levels, detailed ART histories (all previous drugs used and date of any regimen changes); which participants received interventions such as adherence counselling, and how often; attendance of adolescent support groups; missed or late ART clinic visits for treatment collection; and the presence of all HIV-associated opportunistic infections and minor ailments. Participants all had chronic lung disease, and took additional medications while in the study, so risk factors for virological failure may not be generalisable to populations of otherwise healthy CALHIV. At the time of the study, dolutegravir (DTG)-based regimens were not part of standard treatment guidelines; however, DTG is now widely available in both countries, and further investigation is needed on whether the factors associated with non-suppression is the same with DTG-based regimens.

## Conclusions

Early adherence interventions are important to ensure sustained viral suppression. This should start at diagnosis and ART initiation and continue throughout the lifelong treatment period. Enhanced adherence support should be available for all patients with detectable viral load levels while on ART, ideally starting from low viraemia levels, and intensified for those ≥ 1000 copies/mL. This will protect the first line regimen by decreasing the risk of resistance mutations developing and avoid the need for second line regimens that are more expensive, have higher pill burdens and more side effects.

Participants who did switch regimen were much less likely to have viral non-suppression at follow-up. This emphasizes the importance of correct and timely management of unsuppressed viral load. Further research to understand the reasons for low rates of regimen switches from first to second line ART for children and adolescents with virological failure could be in the form of ART record reviews to assess clinical practice, interviews with clinical staff and adherence supporters to determine their knowledge, attitudes, skills and confidence levels in managing adolescents with unsuppressed viral loads, and reviewing data on drug stock-outs, unavailability of viral load testing, and missed or late appointments. The findings of such research can guide policy makers and implementers to take measures to ensure CALHIV have access to the support and treatment they need to sustain viral suppression.

## Data Availability

The datasets used and/or analysed during the current study are not available publicly since the trial was performed at a time when data sharing was not the norm, and therefore participant consent for wider sharing of data was not obtained, but datasets are available from the corresponding author on reasonable request after following institutional requirements.

## Abbreviations

ART: Antiretroviral treatment
ATV/r: Atazanavir and ritonavir
CALHIV: Children and Adolescents living with HIV
CI: Confidence interval
EFV: Efavirenz
FEV1: Forced expiratory volume in one second
HIV: Human Immunodeficiency Virus
INSTI: Integrase Strand Transfer Inhibitor
IQR: Interquartile range
LPV/r: Lopinavir and ritonavir
mL: Millilitre
NNRTI: Non-nucleoside Reverse Transcriptase Inhibitor
NVP: Nevirapine
OR: Odds ratio
PHIA: Population Based HIV Impact Assessment
PI: Protease Inhibitor
TB: Tuberculosis
UNAIDS: Joint United Nations Programme on HIV/AIDS
µL: Microlitre
VL: Viral load
